# Is Concept Appraisal Modulated by Procedural or Declarative Manipulations?

**DOI:** 10.3389/fpsyg.2022.774629

**Published:** 2022-03-22

**Authors:** Sapphira R. Thorne, Joulia Smortchkova, Jake Quilty-Dunn, Nicholas Shea, James A. Hampton

**Affiliations:** ^1^Department of Psychology, City, University of London, London, United Kingdom; ^2^Institute of Philosophy, School of Advanced Study, University of London, London, United Kingdom; ^3^Faculty of Philosophy, University of Oxford, Oxford, United Kingdom; ^4^Philosophy–Neuroscience–Psychology Program, Department of Philosophy, Washington University in St. Louis, St. Louis, MO, United States

**Keywords:** metacognitive judgements, concept appraisal, knowledge, fluency, metacognition, concepts

## Abstract

A recent study has established that thinkers reliably engage in epistemic appraisals of concepts of natural categories. Here, five studies are reported which investigated the effects of different manipulations of category learning context on appraisal of the concepts learnt. It was predicted that dimensions of concept appraisal could be affected by manipulating either procedural factors (spacing of learning, perceptual fluency) or declarative factors (causal knowledge about categories). While known effects of these manipulations on metacognitive judgements such as category learning judgements and confidence at test were replicated, procedural factors had no reliable effects on the dimensions of concept appraisal. Effects of declarative manipulations on some forms of concept appraisal were observed.

## Introduction

Questions surrounding the nature and role of concepts in thought have been at the forefront of research in psychology for many decades. A less explored aspect of concepts is the way in which thinkers assess their own concepts. In a previous empirical study, we have discovered that people evaluate their concepts epistemically. We called this aspect of thinking about concepts “concept appraisal” (Thorne et al., 2021). To explore concept appraisal, we investigated eight dimensions of thinkers’ epistemic evaluations of their concepts (these may be seen in Table 1). The first three dimensions encode how well thinkers understand the concepts they use. To explore understanding we asked participants to evaluate the accuracy of the information they associate with a concept, how much information is contained in a concept, and how well they could explain the concept to someone else. Four other dimensions encode something about the concept itself, and more precisely something about its reliability or dependability as a tool for thinking. To explore this aspect of concept appraisal we asked participants to evaluate how good the concept is for making inductive inferences, how informative the concept is about the objects that fall under its scope, how willing they are to defer to experts regarding its use, and finally how much they think there is to learn about the category. A final dimension of concept appraisal encodes whether thinkers consider the concept to be a useful tool when communicating with others. We have shown that these eight dimensions are reliable (people agree on how to judge different sets of concepts along the dimensions outlined above) and exist for concepts in multiple domains [from natural kinds to social groups to artefacts, for full details see Thorne et al. (2021)].

**TABLE 1:** Nine concept appraisal questions used in Studies 1–5.

Induction	On average, a bird will spend 9% of their day maintaining their feathers. Some birds spend more time maintaining their feathers than others. Suppose that you observe three different members of a particular family of birds spending longer than average maintaining their feathers. How likely is it that the next member of that family of bird will also do so?	No more than chance–A lot above chance	Studies 1–5
Visual induction	If you found out that three different members of a family of birds laid a clutch of eggs that look just like the eggs pictured below, what is the likelihood that another member of that family of birds would also lay a clutch of eggs that look just like these?	No more than chance–A lot above chance	Study 1
Informativeness	How much do you think knowing that an individual bird belongs to a particular family tells us about that bird?	Very informative–Very uninformative	Studies 1 and 3–5
How much to learn	There is more to learn about some categories than others. Starting from scratch, relatively how long do you think it would take to become an expert about the following categories?	Long time–Short time	Studies 1 and 2
Deference	For some things there are experts who could tell you everything you need to know about the category they fall in. Do you think that there are experts for the different families of birds?	Very unlikely–Very likely	Study 1
Explain	How confident do you feel about being able to explain each family of birds to another person?	Very unconfident–Very confident	Studies 1 and 3–5
Accuracy	How sure are you that most of the things you know about each family of birds are true?	Very unsure–Very sure	Study 1
How much do you know	How much do you think you know about each family of birds?	Very unknowledgeable–Very knowledgeable	Studies 1 and 3–5
Communicate	How likely do you think that people can predict what other people have in mind when they talk about these different families of birds?	Very unlikely–Very likely	Study 1

*A selection of these questions were also adapted for each following study. Wording in the table is for Study 1.*

The existence of dimensions of concept appraisal raises the question of whether and how these dimensions could be manipulated. This question is directly relevant to some of the issues that motivated the initial study, and in particular to issues surrounding conceptual engineering in philosophy (Machery, 2017; Thomasson, 2017; Cappelen, 2018), a project that aims at improving and changing our shared understanding of some socially relevant concepts, and issues related to the mechanisms of conceptual change during development (Smortchkova and Shea, 2020). Conceptual change occurs all the time at the level of groups and of individuals, but its mechanisms are still debated, and it is notoriously difficult to achieve in a goal-directed manner. Could the dimensions of concept appraisal discovered in Thorne et al. (2021) play a role in conceptual change? As a first step in answering this question, in the present study we decided to explore which factors could influence the epistemic evaluations of concepts, that is factors that could influence both the subject’s assessment of how well they understand a certain concept, and of their assessment of the reliability of the concept as a good tool for thinking about the world.

As a way of operationalising the issue, we looked at the metacognitive literature exploring the factors that influence people’s evaluations of their own judgements, beliefs, and categories. In this literature a distinction is drawn between procedural or experiential factors (such as procedural fluency in reading) and declarative or theory-based factors (such as the assessment of how well new information fits with the information already possessed by the thinker, Koriat and Levy-Sadot, 1999; Schwarz, 2010; Proust, 2013). Both of these factors have been shown to have an impact on various judgements (such as judgements of truth, judgements of confidence, etc.). We hypothesised that these factors could also have an impact on concept appraisals, namely on judgements of understanding and judgements of reliability. We set out to investigate whether concept appraisals are modulated either by procedural manipulations or by declarative manipulations.

## Overview of the Current Research

Study 1 starts with a much-studied procedural manipulation. It explores the impact of massed versus spaced learning on metacognitive judgements (Kornell and Bjork, 2008; Logan et al., 2012). We adopted an experimental design from Wahlheim et al. (2011) and used natural categories of families of birds. We aimed to test the potential effect on some forms of concept appraisal (judgements about concepts’ understanding, reliability and usefulness for communication) and to replicate the effect of massed learning on the overestimation of one’s future performance after learning (Kornell and Bjork, 2008).

Studies 2 and 3 focus on processing fluency, another procedural factor which is regularly found to affect metacognitive judgements. We used two fluency manipulations: image size and readability of fonts. Study 2 used paintings in two different categories (expressionist and minimalist) and manipulated their size to induce experiences of fluency and disfluency. Size has been shown to influence metacognitive judgements (Undorf et al., 2017). Study 3 used verbal descriptions for fictional types of ants [adapted from Rehder and Hastie (2004)] and used font size to induce experiences of fluency and disfluency (Kaspar et al., 2015). In both studies the impact of fluency on predictions of one’s performance and on concept appraisal is explored.

Studies 4 and 5 turn to declarative factors. They test the possible impact of information about the structure of the newly learnt categories on concept appraisal. Here we adapted a design from Rehder and Hastie (2004) and explored the influence of the structure of the category (as having properties that are produced by a common cause, as having properties inducing a common effect, as having properties related in a causal chain, or as having properties with no causal relations as a control condition) on a series of questions about the categories. Do causal beliefs about the newly learnt categories influence the ways the concepts are appraised epistemically?

## General Method

Inspired by research on category learning judgements (Jacoby et al., 2010), we adopted a method that has been used to manipulate various types of metacognitions associated with category learning (Wahlheim et al., 2011). An adaptation of this general method was used in Studies 1–5. These studies involved three main parts: A study phase, a concept appraisal phase, and a test phase.

The *study phase* was presented on either *Microsoft Visual Basic version VB15x* (Studies 1, 2, and 5) or *Qualtrics* (Qualtrics, 2018, Provo, UT, United States) (Studies 3 and 4). During the study phase, 5–12 exemplars from several categories were presented randomly to participants. All exemplars were presented on a computer monitor against a white background. During the study phase, participants saw either visual (Studies 1 and 2) or written (Studies 3–5) exemplars, one at a time, together with the corresponding category name. After each exemplar was presented, the same exemplars were presented again in another random order. Following the second presentation of exemplars participants made predictions regarding the number of subsequent exemplars they would be able to classify correctly on a classification task, a measure known as category learning judgements (CLJs).

During the *concept appraisal phase*, participants completed several questions assessing dimensions of concept appraisal for each of the different categories studied. The full list of concept appraisal questions used throughout these studies is presented in Table 1. For Study 1, the induction dimension used in Thorne et al. (2021), was expanded into verbal and visual induction dimensions, leading to nine questions in all.

The *test phase* was presented using the same programme as the study phase. Participants were shown between 4 and 10 exemplars from each category (depending on the Study), at least half of which had not been seen previously, and had to decide which category the exemplar belonged to. After each classification, participants provided a confidence judgement about their classification on a scale of 1 (very unconfident) to 5 (very confident). Participants were unable to change their classification judgements after making a selection. All exemplars appeared in a random order. Participants were given as much time as they needed to complete the concept appraisal questions and the test phase.

## Study 1

In the first study we wanted to explore whether the spacing effect shown in the previous metacognition literature (Kornell et al., 2010) would influence the dimensions of concept appraisal. This is a metacognitive manipulation, where subjects presented with new categories to learn tend to be more confident in the effectiveness of their learning when the categories are presented in a massed way (Kornell and Bjork, 2008). This confidence, however, does not track their actual performance, as it has been shown that spaced learning is often more conductive to better recall (Son, 2004; Kornell and Bjork, 2008; Wahlheim et al., 2011). We wanted to test the hypothesis that the way in which categories are learned could influence subjects’ judgements about the dimensions of concept appraisal, with massed learning leading to more confident judgements, and greater ratings for understanding, reliability and communication.

### Method

#### Participants

Forty-three participants (24 Female, 18 Male, and 1 unspecified) recruited through an opportunity sample through the recruitment pool at City, University of London, participated in this study in exchange for a small monetary reward. Two participants whose performance fell more than two standard deviations below the mean (20% or less correct) were excluded from the study leaving *N* = 41 (Age 18–54; *M*_Age_ = 26.34). Sample size was sufficient to provide a power of 0.87 to detect a medium sized effect (*d* = 0.5).^1^

#### Design and Materials

To select stimuli for the study, we selected ten exemplars from bird families used by Wahlheim et al. (2011) from images on www.whatbird.com. All presented images were 450 px by 450 px. Initially 12 bird families were selected (*Chickadees*, *Finches*, *Flycatchers*, *Grosbeaks*, *Jays*, *Orioles*, *Sparrows*, *Swallows*, *Thrashers*, *Thrushes*, *Vireos*, and *Warblers*). A pre-test (*N* = 33) obtained ratings of within-family similarity for each family of birds on scales of 0 (extremely dissimilar) to 100 (extremely similar) (see Supplementary Materials for further details). To ensure that the families of birds selected were relatively equal in terms of similarity, the six families of birds that received the most medium ratings (*Jays*, *Orioles*, *Sparrows*, *Swallows*, *Thrushes*, and *Vireos*) were selected for use in this study. Ten exemplars from each of these bird categories were selected as stimuli for this study, five or which were presented in the study phase. There were some marginal differences between the six families of birds in terms of similarity [*F*(5, 160) = 2.30, *p* = 0.06, η^2^ = 0.07]. The 30 exemplars used for the study phases were presented one at a time for 4 s in two blocks of 15 trials. These exemplars were then each presented a second time for 2 s each in another two blocks of 15 trials. Exemplars were randomised between blocks.

A 2 factor (Study: Massed vs. Spaced) within-subjects design was implemented whereby participants learned six categories of birds, three of which were presented in a massed sequence and three in a spaced sequence. In the study phase, for massed blocks, participants were presented with study items from three categories, with five from the first, then five from the second and then five from the third. In spaced blocks, the 15 birds from the three categories were randomly ordered. Which bird families were assigned to massed versus spaced conditions was balanced across participants, and the type of study presented first in the experiment was also counterbalanced. The blocks were presented in one of two orders MSMS (*N* = 22) or SMSM (*N* = 21), where *M* refers to blocks where the categories were presented in a massed fashion, and S referred to blocks where the categories were presented in a spaced fashion. Blocks 3 and 4 used the same categories as Blocks 1 and 2, respectively. Exemplars were presented in a new random order for each participant. In the test phase, all ten exemplars from each category of birds were presented to participants for naming.

#### Procedure

The procedure for this study followed the general method. During the study phase, exemplars were presented in either a massed or spaced fashion; exemplars from the six different categories of birds were presented in four blocks of fifteen trials. Following this study phase, participants made CLJs and answered questions about the eight dimensions of concept evaluation (see Table 1) for each category of birds. Finally, participants completed the test phase in which the 30 old together with 30 new exemplars appeared in a random order, and had to be assigned to the correct category. The test phase consisted of 60 trials with a break halfway through. Ethical approval for all studies in this manuscript was obtained from the Psychology Research Ethics Committee, City, University of London.

### Results

The aim of this analysis was to determine the effect that the massed and spacing manipulation had on how participants appraised the eight dimensions of concept appraisal. First, we conducted analyses to determine the effectiveness of the manipulation on participants’ judgements. If our manipulation was effective, then, consistent with previous literature, in their CLJs participants would overestimate their future performance for massed categories but not for spaced categories.

#### Classification Performance

**FIGURE 1:**
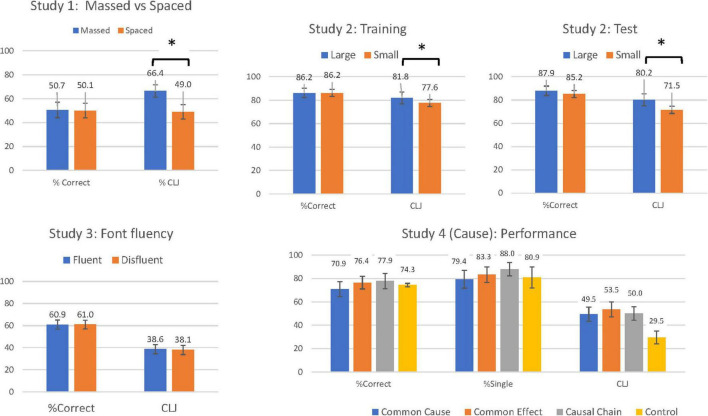
Percent correct and estimated performance or category learning judgements (CLJ) in Studies 1–4. *Significant at *p* < 0.05.

To examine the accuracy of participants’ predictions, in this and subsequent studies, CLJs (estimated performance) for massed and spaced categories were converted into a percentage and compared to the percentage of exemplars from these same categories that were correctly classified. Comparison of estimated (CLJ) and actual performance was treated as a within-subjects factor labelled Performance. The results are shown in the first panel in Figure 1. A 2 (Order: Massed first vs. Spaced first) × 2 (Performance: CLJ/Estimated vs. Actual) × 2 (Study: Massed vs. Spaced) Mixed ANOVA revealed a main effect of Performance [*F*(1, 39) = 6.23, *p* = 0.02, η_p_^2^ = 0.14], indicating that participants overestimated their future performance on the task (CLJ: 57.7% vs. Actual performance: 50.4%). Further, there was a main effect of Study, with estimated and actual performance, taken together, higher for massed categories (*M* = 58.5%, SD = 14.9) than for spaced categories [*M* = 49.6%, SD = 14.3, *F*(1, 39) = 11.76, *p* = 0.001, η_p_^2^ = 0.23]. The benefits of massed categories were qualified by a significant interaction between Study and Performance [*F*(1, 38) = 17.68, *p* < 0.001, η_p_^2^ = 0.31]. Unlike Wahlheim et al. (2011), who had a somewhat different method, participants only overestimated their performance when the categories were presented in a massed fashion [*t*(40) = 4.62, *p* < 0.001, *d* = 0.77] and not when categories were presented in a spaced fashion [*t*(40) = 0.27, *p* = 0.79, *d* = 0.05].

#### Confidence Ratings

**FIGURE 2:**
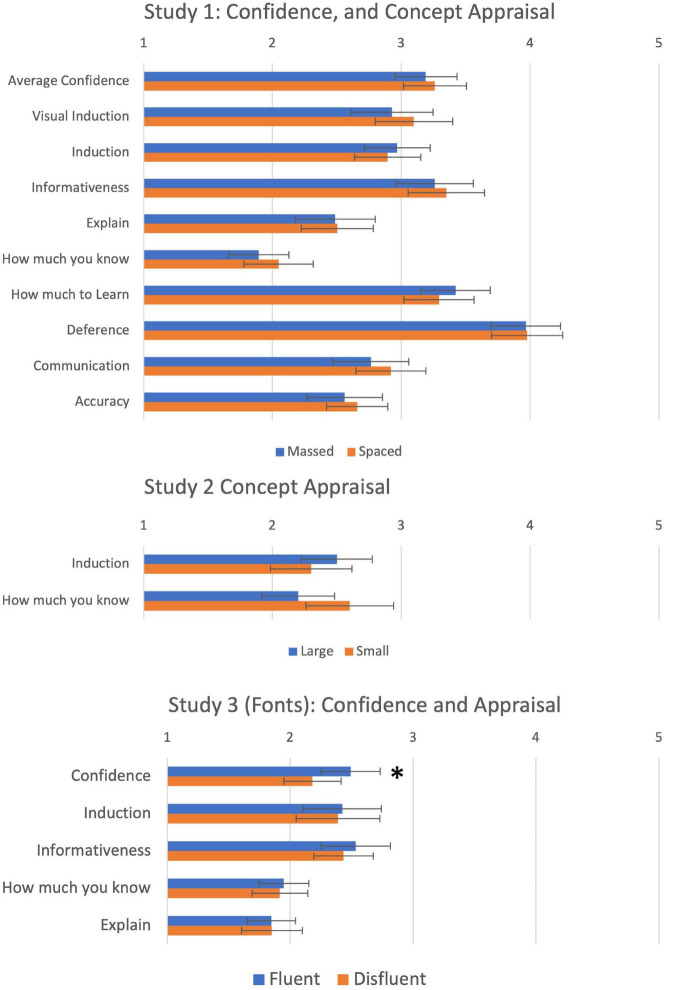
Average confidence at test, and concept appraisal for Studies 1–3. *Significant at *p* < 0.05.

At test, participants rated confidence in each of their categorisation judgements on a scale from 1 to 5. Mean confidence was calculated for each condition. Means are shown as the first row in the top panel of Figure 2. A 2 (Order: Massed first vs. Spaced first) × 2 (Stimuli: Old vs. Novel) × 2 (Study: Massed vs. Spaced) mixed ANOVA revealed that participants were more confident classifying exemplars they had seen in the study phase (*M* = 3.31, SD = 0.74) than exemplars that were novel [*M* = 3.15, SD = 0.74; *F*(1, 39) = 15.93, *p* < 0.001, η_p_^2^ = 0.29]. However, there was no significant difference between massed and spaced categories [*F*(1, 39) = 0.96, *p* = 0.33, η_p_^2^ = 0.02], indicating that the manipulation did not have an effect on post-dictions. There were no other main effects or higher order effects from this analysis.

#### Concept Appraisal

Having established that our manipulation was effective, we next tested whether the manipulation influenced the nine dimensions of concept appraisal. Results are also shown in the top panel of Figure 2. A 2 (Order: Massed first vs. Spaced first) × 2 (Study: Massed vs. Spaced) MANOVA was conducted with the nine questions on the metacognitive questionnaire. The MANOVA revealed no main effect of either order [λ = 0.25, *F*(9, 31) = 1.14, *p* = 0.37, η_p_^2^ = 0.25] or Study [λ = 0.33, *F*(9, 31) = 1.72, *p* = 0.13, η_p_^2^ = 0.33], and no significant interaction [λ = 0.29, *F*(9, 31) = 1.37, *p* = 0.24, η_p_^2^ = 0.29] (Wilks Lambda reflects the proportion of variance *not* attributable to effects). Thus, we found no evidence that a massed vs. spaced manipulation that succeeded in modifying CLJs had any influence on either confidence judgements, or judgements of concept appraisal.

How strong was the evidence for the null hypothesis here? Bayesian statistics for MANOVA are complex (Press, 1980), so a simplified approach was taken. Each of the nine *F* ratios for the univariate effects of the Study factor on the nine dependent variables was used to calculate a Bayes Factor using JASP (2021). Values below 1 indicate strength of support for the null hypothesis. Values ranged from 0.18 to 1.6, with a median of 0.31. Seven of the nine were below 1, suggesting greater support for the null than for the alternate hypothesis.

### Discussion

Contrary to Wahlheim et al. (2011) subjects tended to overestimate their CLJs for massed rather than for spaced presentations of the categories. This is likely owing to the difference in the methods used. Indeed, our result is consistent with the known influence of massed presentation on learning of new categories where subjects think that they learn more when the stimuli are presented in a massed way (Kornell and Bjork, 2008; Kornell et al., 2010): we have found that the spaced vs. massed learning manipulation leads to increased estimates of future performance (CLJ) when the items are presented in a massed way, even when these CLJs do not track actual performance. This effect is plausibly due, at least in part, to processing fluency: subjects experience more fluency in the massed learning condition than in the spaced learning condition that is felt to be disfluent (Kornell et al., 2010; Wang and Xing, 2019). No increase in confidence was observed for postdictive judgements nor for the eight dimensions of concept appraisal. While this experiential manipulation of the way concepts are presented had an impact on the metacognitive evaluation of future performance, it appears not to have had an impact on the evaluation of the concepts themselves for their reliability or on the evaluation of the learner’s understanding of the concepts.

## Study 2

The second study used image size (Undorf et al., 2017) to introduce a fluency manipulation in the classification of two schools of 20th Century paintings. The aims were similar to Study 1, to assess the effect of this manipulation on metacognitive and appraisal judgements.

### Method

#### Participants

Eighty-nine participants (50 Female, 40 Male, and 3 unspecified) aged between 18 and 76 (*M*_Age_ = 34.91) were recruited at an Institute of Philosophy Public Engagement event (“Self Impressions”) at the Tate Modern in London, and completed the procedure. Sample size (limited by attendance at the event) provided power of 0.64 for a medium-sized effect (*d* = 0.5). Because of the need to keep the study time to a minimum for our volunteers, we focussed on just two concept appraisal dimensions *Induction* and *How Much to Learn*, as representing appraisals of the dependability of the classification.

#### Design, Procedure, and Materials

**FIGURE 3:**
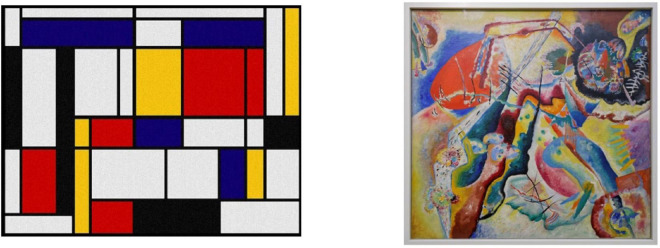
An example of a Minimalist painting (left): “Composition en rouge, jaune, bleu et noir” Piet Mondrian, and an Expressionist painting (right): “Painting with red spot” Wassily Kandinsky.

Participants completed the study implemented in *MS Visual Basic* on a 14-inch laptop. The design of this study followed the general method using categories of paintings. Participants learnt to distinguish between two different styles of painting: Minimalism and Expressionism. An example of each style of painting can be seen in Figure 3. Fifteen exemplars from each style of painting were selected as stimuli for this study; ten from each category were presented in the study phase, and the remaining five were used for the test phase.

A between-subjects design was implemented. Participants were randomly assigned conditions where the images presented in the study phase were either large (60% of the screen width; *N* = 43) or small (15% of the screen width; *N* = 46). First was a study phase where participants were presented with an image of a painting and were given 5 s to decide whether the painting was a minimalist or an expressionist style of painting. They were provided with feedback about the correct classification after each trial. Participants completed one block of 20 trials. Following the study phase participants gave CLJs (predicting how well they would do in the test phase) and a further judgement about how well they thought they had done in the study phase, both on scales from 0 to 100%. The test phase occurred directly after the study phase. The test phase had the same structure as the study phase, using new images, but no feedback was given after each trial, and images were presented at intermediate size.

After the test phase, concept appraisal questions were completed on a pencil and paper questionnaire. Alongside the two questions on *Induction* and *How Much To Learn* in Table 1, participants also indicated their knowledge of art on a scale from 1 (not very much) to 5 (a lot), and their prior knowledge of minimalism and expressionism with binary questions (yes or no).

### Results

As with our previous study, the aim of this analysis was to determine the effect that a metacognitive manipulation – in this case a manipulation of processing fluency via the size of the images – had on the two tested dimensions of concept appraisal. We first conducted analyses to determine whether our fluency manipulation was effective to replicate the known effect on CLJs. If our fluency manipulation was effective, then, participants presented with larger images during the study phase would give higher estimations of their performance at test than participants presented with smaller images. We predicted actual performance to be the same across fluency conditions.

#### Predicted and Measured Classification Performance – Training

Percentage Correct in training and judgements of how well they had performed in training are shown in the second panel of the top row in Figure 1, while Percent Correct at test and Predictive CLJs for the test session are shown in the third panel. For the training session, a 2 (Size: Large vs. Small) × 2 (Performance: CLJ/Estimated vs. Actual) mixed ANOVA revealed that estimates of performance were higher when images were large than when images were small [*F*(1, 87) = 4.02, *p* = 0.048, η_p_^2^ = 0.044]. Participants generally underestimated their performance on the task [79.72 vs. 86.18; *F*(1, 87) = 36.88, *p* < 0.001, η_p_^2^ = 0.30], but this effect was qualified by a significant interaction [*F*(1, 87) = 4.02, *p* = 0.048, η_p_^2^ = 0.044]. As predicted, participants presented with large images (*M* = 81.84, SD = 13.08) thought they had done better than participants presented with small images (*M* = 77.61, SD = 17.41) although actual performance was the same.

#### Predicted and Measured Classification Performance – Test

Similar analysis was run for predictions of test performance (CLJ) and actual test performance (see top right panel, Figure 1). Underestimation of performance was more extreme [*F*(1,87) = 43.50, *p* < 0.001, η_p_^2^ = 0.33], with participants predicting 76% correct as against performance of 87%. The interaction of Performance with Size approached significance [*F*(1,87) = 3.37, *p* = 0.07, η_p_^2^ = 0.33]. Size of the paintings had a significant effect on CLJs [*t*(87) = 3.04, *p* = 0.003], but not on percent correct at test (see Figure 1).

#### Concept Appraisal Questions

To test for the effect of the fluency manipulation on concept appraisal (*Induction* and *How Much To Learn*), a between-subjects MANOVA was conducted, and results are shown in the middle panel of Figure 2. As in Study 1, the MANOVA was not significant [λ = 0.96, *F*(2, 92) = 2.17, *p* = 0.12, η_p_^2^ = 0.045], indicating that our fluency manipulation had no effect on how subjects assessed the concepts for the two tested dimensions of concept appraisal: *Induction* and *How Much To Learn*. Bayes Factors for the two univariate ANOVA were 0.32 and 0.26, indicating weak evidence in favour of the null.

### Discussion

In Study 2 we found an effect of procedural fluency (in this case manipulated via the size of the images) on subjects’ estimations of both their past and future performance: subjects who saw larger images thought they had done better, and predicted better performance than those seeing smaller images, even if their estimates did not track actual performance.

We did not find an effect on the two tested dimensions of concept appraisal. Fluency did not show any influence on subjects’ judgements about how good the two categories would be for making inductions nor for how much there is to know about the two categories. One possibility is that concept appraisal is related to the subjects’ propositional knowledge and not to the visual information related to the concept in question. To explore this option, in the next study we used lists of written descriptions that described categories instead of using visual categories directly. These stimuli were more similar to the ones we used in our original study on concept appraisal (Thorne et al., 2021).

## Study 3

In Study 3 we changed the type of stimuli from visual categories (pictures) to verbal categories (lists of features) and we applied a fluency manipulation to the stimuli by varying the type of font used: one font was easy to read, whereas the other font made reading more difficult. Previous metacognitive research showed that this manipulation can influence a variety of metacognitive assessments (Kaspar et al., 2015) including judgements of learning (Yang et al., 2018). We wanted to see whether this fluency manipulation could also influence the way in which subjects evaluated the newly learnt concepts for a representative four of the eight original dimensions of concept appraisal (Table 1).

### Method

#### Participants

To increase the power of our between-subjects design, 125 participants were recruited for an online study through Prolific Academic in exchange for a small monetary award. Five participants withdrew part-way through the study, two participants indicated using additional aids to complete the task, and four participants performed more than two standard deviations below the mean. These participants were all excluded from the final analyses. The final sample for this study consisted of 114 participants (71 Female, 43 Male) aged between 18 and 65 (*M*_Age_ = 33.74). Power was estimated as 0.77 for a medium-size effect (*d* = 0.5).

#### Design and Materials

The design of this study followed the general method. Participants learned two categories of fictional ants (*Kehoe ants* and *Victoria ants*) which were adapted from the categories used by Rehder and Hastie (2004). As illustrated in Table 2, the prototype for each category was composed of six attributes, with opposing values characteristic of each category of ant. Exemplars were constructed from each prototype by switching either one or two of the six attribute values to that of the opposite category. Twelve exemplars were created for each of the categories; eight to be presented in the study phase, and four being reserved for the test phase, where they were presented alongside four exemplars previously seen in the study phase. To increase power further, we chose to focus on just 4 of the 9 dimensions used in Study 1, two representing understanding, and two reliability.

**TABLE 2:** Features associated with each category (Kehoe ants and Victoria ants) (Studies 3–5).

Feature	Kehoe ants	Victoria ants
(1)	Blood that is very high in iron sulphate	Blood that is very low in iron sulphate
(2)	A hypoactive immune system	A hyperactive immune system
(3)	Blood that is very thin	Blood that is very thick
(4)	Higher than average body weight	Lower than average body weight
(5)	Secrete a fluid that is slightly alkaline	Secrete a fluid that is slightly acidic
(6)	A short lasting flight response to flee from potential predators	A long lasting flight response to flee from potential predators

*In Studies 4 and 5, feature 5 was removed and feature 4 was presented last in order to make the task easier for participants.*

**FIGURE 4:**
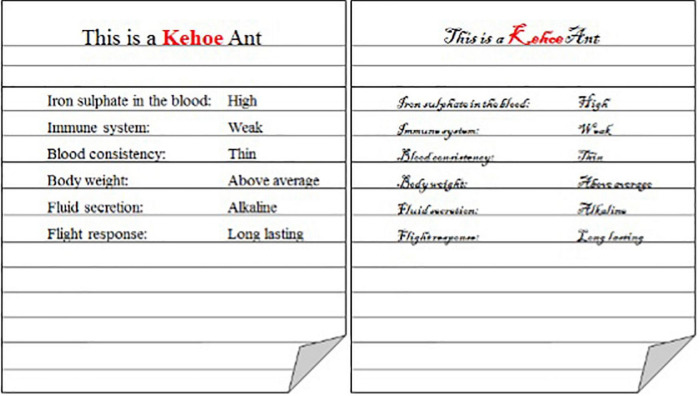
Example exemplar in the fluent **(left)** and disfluent **(right)** font conditions.

A between-subjects design was implemented. Participants were randomly assigned to one of two groups where they learnt the characteristics of the categories, and the category exemplars, either in a 12 pt Times New Roman font (fluent; *N* = 60) or a 12 pt Blackadder ITC font (disfluent; *N* = 58; see Figure 4).

#### Procedure

To begin, participants were presented with a backstory [adapted from Rehder and Hastie (2004)] for the two fictional categories they were going to learn about (see Supplementary Materials for exact wording). Participants completed two blocks of 16 learning trials. As with Study 1, each exemplar was presented twice; once in the first block for 6 s and once in the second block for 3 s. Exemplars were presented in a random order for each participant. Participants then provided CLJ ratings, and evaluated their new concepts along four dimensions of appraisal selected to represent understanding (*How much you know* and *Explain*), and reliability (Induction and Informativeness, see Table 1), before completing the test phase, which included giving confidence judgements on each trial. The entire study was presented on Qualtrics.

### Results

As with the previous studies, we first assessed the effectiveness of the fluency manipulation. We anticipated that if the fluency manipulation was effective then participants assigned to the fluent condition would predict better performance on the task in their CLJ, than participants assigned to the disfluent condition.

#### Classification

As with Study 1, CLJ were converted into percentages to allow for a more standardised comparison between CLJs and actual performance. Results are shown in the lower left panel of Figure 1. A 2 (Study: Fluent vs. Disfluent) × 2 (Performance: CLJ/Estimated vs. Actual) Mixed ANOVA revealed no main effect of fluency condition [*F*(1, 111) = 0.01, *p* = 0.81, η_p_^2^ < 0.001]. Although, participants greatly underpredicted their performance on the task [38.36 vs. 61.07%, *F*(1, 111) = 154.49, *p* < 0.001, η_p_^2^ = 0.58], this underconfidence was not moderated by a higher order interaction with fluency condition [*F*(1, 111) = 0.03, *p* = 0.85, η_p_^2^ < 0.001]. This result differs from the findings in Studies 1 and 2 and indicates that the fluency manipulation may not have been effective in this Study on CLJs.

#### Confidence Judgements

Average Confidence judgements across trials at test were calculated for each condition and are shown as the first row of the bottom panel in Figure 2. A 2 (Study: Fluent vs. Disfluent) × 2 (Stimuli: Old vs. Novel) mixed ANOVA revealed that participants in the fluent condition (*M* = 2.49, SD = 0.92) were found to be more confident in their performance at test than participants in the disfluent condition [*M* = 2.16, SD = 0.86; *F*(1, 112) = 3.96, *p* = 0.049, η_p_^2^ = 0.03]. There was no main effect of Stimuli [*F*(1, 112) = 0.10, *p* = 0.75, η_p_^2^ = 0.001], and no significant interaction between the two independent variables [*F*(1, 112) = 1.31, *p* = 0.26, η_p_^2^ = 0.01]. Therefore, whilst fluency did not influence people’s prediction of their future performance, there is some evidence that it affected people’s confidence in the test phase, suggesting that the verbal manipulation may have influenced a different of metacognitive process than visual manipulations.

#### Concept Appraisal

Having established the effectiveness of fluency for manipulating confidence, we next tested whether the manipulation influenced subjects’ appraisal of concepts. Results are shown in the lower panel of Figure 2. A between-subjects MANOVA on the four evaluation questions revealed no main effect of fluency condition [λ = 0.997, *F*(4, 115) = 0.09, *p* = 0.98, η_p_^2^ = 0.003]. Bayes Factors for the null were 0.20–0.21 for the four dimensions, indicating weak support for the null. Thus, once again we found no evidence that fluency manipulations, which are strong enough to replicate known effects on metacognitive judgements, have any effect on subjects’ appraisal of concepts.

### Discussion

In the study we applied a manipulation of processing fluency by changing the font of the texts the subjects read. We used texts describing fictional natural categories consisting of species of ants (Rehder and Hastie, 2004) instead of visual images to verify whether the type of stimuli could make a difference to the effect of fluency on subjects’ evaluations of concepts. We found a small influence of fluency on confidence judgements regarding performance. We did not find an influence on CLJs nor on the eight dimensions of subjects’ evaluations of the concepts.

Having now reported three studies exploring whether fluency manipulations can have an impact on how subjects assess their own concepts, we hypothesise that concept appraisal might not be influenced by experiential and procedural factors such as a feeling of fluency.

As a result, we turned from procedural to declarative factors. We decided to explore whether manipulations of beliefs associated with the concepts would have an impact on concept appraisal. This is the aim of Studies 4 and 5: here we manipulate subjects’ explicit beliefs about the categories and explore whether this has an impact on how they assess their concepts for understanding, reliability, and communication.

## Study 4

In this study we manipulated subjects’ explicit beliefs about new categories. To do so we adapted a design from Rehder and Hastie (2004) where they compared different ways of structuring a conceptual representation via graphs. They used four conditions: properties having a common cause, properties having a common effect, properties related in a causal chain, and no relation as the control condition. They found that having a causal structure had an influence on subjects’ tendency to use the concept for inductive generalisations (with the common cause structure having the strongest effect). Induction is one of the dimensions of concept appraisal we identified in previous experiments (Table 1; Thorne et al., 2021). In Study 4 we wanted to explore whether adding an explicit theoretical belief about the categories’ structure could have an impact on how subjects evaluated the new concepts along the same four dimensions of concept appraisal.

### Method

#### Participants

Two hundred and one participants recruited for an online study through Prolific Academic participated in this study in exchange for a small monetary award. Sample size in this study and the next reflected the increase in the number of between-subject conditions from 2 (in Study 3) to 4 in Studies 4 and 5. Two participants withdrew part-way through the study, three participants indicated using additional aids to complete the task, and eight participants were identified as extreme outliers in the test phase (more than two standard deviations below the mean). These participants were all excluded from analyses. The final sample for this study consisted of 188 participants (114 Female, 73 Male, and 1 unspecified) aged between 18 and 73 (*M* = 35.48). Power was estimated at 0.82 for a medium-size effect (*f* = 0.25).

#### Design and Materials

The design for this study followed the general method and used the same two categories of ants used in Study 3. However, to make the task easier for participants only five features were used when creating exemplars. Again, twelve exemplars with one or two swapped attribute values were created from the prototypes of each of these two fictional categories of ants; eight were presented in the study phase, the remaining four exemplars were reserved for the test phase, alongside four exemplars previously seen in the study phase. The set of eight exemplars had three with one distortion, and five with two distortions, while the four new items in the test phase had two with one distortion and two with two distortions. The 16 exemplars used for the study phase were presented one at a time for 6 s in a single block of 16 trials. These exemplars were then each presented a second time for 3 s each in another block of 16 trials. Exemplars were presented in a random order.

**FIGURE 5:**
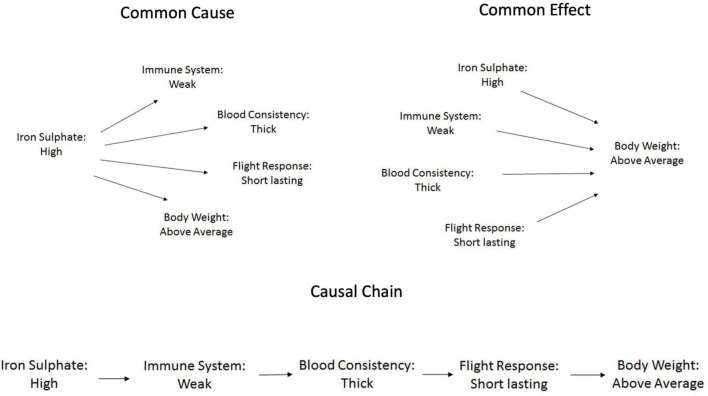
Causal structures for the three causal conditions for Kehoe ants (Studies 4 and 5).

A between-subjects design was implemented; participants were randomly assigned to one of four conditions: three causal conditions (Common Cause, Common Effect, or Causal Chain) and a control condition. These were the same conditions used by Rehder and Hastie (2004). The assignment to these conditions referred to the type of information provided to participants in the study phase. Prior to the presentation of exemplars, all participants, regardless of condition, were presented with the prototype of both types of ants. Participants assigned to one of the three causal conditions were also informed that the characteristics associated with the two types of ants were causally related to each other and were provided with further information about the causal structure, accompanied by an image of the causal structure (Figure 5).

Participants assigned to the “common cause” condition (*N* = 46) were informed that the first feature (*level of iron sulphate in the blood*) caused the emergence of the other four features. Those in the “common effect” condition (*N* = 51) were told that the last feature (*body weight*) was caused by the other four features. Finally, participants in the “causal chain” condition (*N* = 48) received information that the five characteristics form a causal chain so that each one influences the next one along the chain starting with the first feature (*level of iron sulphate in the blood)* and ending with the last feature (*body weight*). Each causal relationship was described by a single sentence. In the control condition (*N* = 43) there was no information about relations between features. Table 3 describes the causal relationships for each category of ant. Participants received the prototype and information about the causal structure for both categories of ants. The order in which the two ants were presented was counterbalanced across participants; 97 saw Kehoe ants first, 91 saw Victoria ants first. Then participants completed the study and concept appraisal phases. With the exception of a new set of exemplars these two phases were identical to Study 3.

**TABLE 3:** The casual structure for Kehoe ants (Studies 4 and 5).

Cause	Effect	Description
F1	F2	Iron tends to inhibit the expression of an important molecule, thus weakening the immune system
	F3	Excess iron in the blood can lead to the production of extra red blood cells which thicken the blood
	F4	The excess iron in the blood can interfere with the production of hormones which support a long flight response
	F5	The extra iron can mean that the ant consumes more nutrients resulting in higher body weight
F2	F3	A weakened immune system results in fewer blood proteins, which prevent blood platelets from clumping, leading to thicker blood
	F5	A weakened immune system can affect an inhibitory system that controls eating, leading to more body weight
F3	F4	Thick blood results in a low increase of hormones that mediate the flight response, meaning that the flight response is short
	F5	Thick blood can result in an increase in chemicals that regulate the storage of fat resulting in an increased body weight
F4	F5	Ants with a short flight response tend to deplete their resources rapidly and consume more food resulting in an increase in body weight

*Victoria ants had the same causal structure and very similar wordings.*

In the test phase, participants completed two tasks. In the first task (the Exemplar Classification Task), participants made classification and confidence judgements of 16 exemplars, based on four “novel” exemplars from each category, not previously seen in the study phase, and four “old” exemplars from each category, which they had seen in the study phase. Following this, as an additional test of classification confidence, participants completed a second task (the Single Feature Task) that provided participants with ten new exemplars in random order. Each of these exemplars reported a single feature that was typical of either one category or the other. Participants made classification and confidence judgements for each of these exemplars.

#### Procedure

Participants were first presented with the same backstory about the two fictional categories presented in Study 3. When participants turned the page, they were shown a table of the prototypical features of one of the two fictional categories and were given as much time as they needed to study the table. Participants in the three causal conditions were also presented with further information about the causal relationships between the five features. Participants then received the corresponding information about the second fictional category. Participants in the control condition were only presented with the table of features associated with each category of ant. Following presentation, participants completed the study, metacognitive and appraisal, and test phases. The study was completed on Qualtrics.

### Results

To test the effectiveness of the causal category manipulation we assessed the differences between the four conditions in performance during the test phase. We anticipated that if the causal category manipulation was effective then participants would rely upon the causal structure information given in the first part of the study during the test phase, leading to differences in performance.

#### Exemplar Classification Task

Percent Correct and CLJs transformed to percentages were calculated for each condition and are shown in the lower right panel of Figure 1. A 4 (Causal Condition: Common Cause vs. Common Effect vs. Causal Chain vs. Control) × 2 (Performance: Actual vs Estimated/CLJs) mixed ANOVA was conducted. There was no main effect of causal condition [*F*(3, 183) = 0.78, *p* = 0.51, η_p_^2^ = 0.01]. However, there was a main effect of actual (Mean 74.9%) vs. estimated (Mean 50.2%) performance, indicating that participants underestimated performance on the task [*F*(1, 183) = 228.89, *p* < 0.001, η_p_^2^ = 0.56]. Crucially there was no significant interaction between the two independent variables [*F*(3, 183) = 0.52, *p* = 0.67, η_p_^2^ = 0.01]. These results provide very little indication that that the causal manipulation had an effect on individuals’ metacognitive beliefs.

#### Single Feature Task

Percent correct was also calculated for the single feature task, and results are also shown in Figure 1. A between-subjects ANOVA revealed no main effect of causal condition on accuracy [*F*(3, 184) = 1.23, *p* = 0.30, η_p_^2^ = 0.02], suggesting again that there were very limited differences in actual performance between the four conditions.

#### Confidence Judgements

**FIGURE 6:**
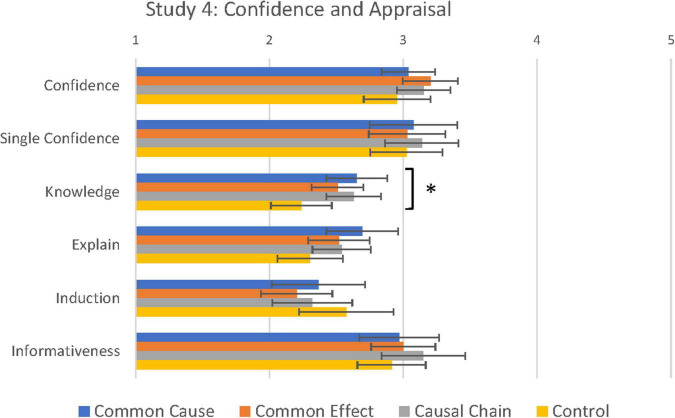
Confidence and concept appraisal as a function of causal condition in Study 4. *Main effect of condition significant at <0.05, see text.

We next tested differences between causal conditions in confidence judgements given during the test phase. Mean confidence judgements for Study 4 are to be found in Figure 6, together with the Appraisal judgements (see next section). A 4 (Causal Condition: Common Cause vs. Common Effect vs. Causal Chain vs. Control) × 2 (Stimuli: Old vs. Novel) Mixed ANOVA revealed no differences between causal conditions in confidence judgements [*F*(3, 184) = 1.04, *p* = 0.38, η_p_^2^ = 0.02]. There was a main effect of Stimuli [*F*(1, 184) = 20.78, *p* < 0.001, η_p_^2^ = 0.10]. Strangely, participants were more confident with novel exemplars than previously seen exemplars (Old: 3.07 vs. Novel: 3.19), but this was not qualified by a significant interaction with causal condition [*F*(3, 184) = 0.91, *p* = 0.44, η_p_^2^ = 0.02]. These results suggest that causal condition may have only limited influence on confidence judgements. In support of this conclusion, the Single Feature task also showed no differences between conditions in confidence in classification performance [*F*(3, 184) = 0.42, *p* = 0.74, η_p_^2^ = 0.01].

#### Concept Appraisal

More crucial to our aims, we next tested whether the causal manipulation had an effect on participants’ appraisal of how well they understood the categories and how reliable they were (see means in Figure 6). The between-subjects MANOVA on the four evaluation questions revealed a marginal main effect of causal condition [λ = 0.10, *F*(12, 549) = 1.60, *p* = 0.09, η_p_^2^ = 0.04]. Exploratory univariate analyses revealed a significant difference between conditions in Knowledge (*How Much You Know)* [*F*(3, 184) = 3.75, *p* = 0.01, η^2^ = 0.06]; in *post hoc* comparisons, participants assigned to the three causal conditions (CC: *M* = 2.70, SD = 0.79, compared to control *p* = 0.005; CE: *M* = 2.55, SD = 0.69, *p* = 0.03; CChain: *M* = 2.66, SD = 0.72, *p* = 0.006) felt more knowledgeable about the two categories of ants than participants assigned to the control condition (*M* = 2.22, SD = 0.077). Participants who were assigned to the three causal conditions and received further information about the two categories of ants were aware of their greater knowledge of the categories. No other *post hoc* tests were found to be significant. There were no significant differences between conditions for the two reliability judgements (*Informativeness: p* = 0.48 and *Induction*: *p* = 0.63).^2^ These results indicate that whilst the subjects’ assessment of how well they understood the concept could be manipulated by providing further information about the category, the subjects’ assessment of how reliable the concepts are may be more difficult to manipulate.

### Discussion

Studies 1–3 showed no effect of fluency manipulations on dimensions of concept appraisal. In Study 4 we tested a manipulation of beliefs associated with new categories (new species of ants) to explore whether these beliefs relating to the structure of two distinct categories could have an impact on how subjects appraised their concepts. We found that having declarative information about the causal structure of the category made subjects rate the categories higher on the dimension *How Much You Know* (in all three causal conditions). However, having new information had no impact on their judgements about the reliability of the new concept. Perhaps presenting participants with several exemplars that contradicted the causal structure of the category of ants reduced the legitimacy of the category. We test this in Study 5.

## Study 5

In the final study instead of using two categories of ants, we only used one. We presented different groups of participants with three types of causal relations (common cause, common effect, and causal chain) and had a control group that didn’t see any type of causal relation. We added a quiz to ensure the features and causal structures were understood, so that it could be taken into account in appraising the categories. To widen our exploration of the influence of causal knowledge on concept appraisal we also expanded the list of appraisal questions from the previous studies to a total of 15 dimensions. We were interested to discover if other diverse metacognitive questions would show an influence of the manipulation of causal structure.

### Method

#### Participants

Two hundred and nine participants were recruited for an online study through Prolific Academic. Eight participants withdrew before the concept appraisal phase of the study and six participants answered more than one question incorrectly during the feature and/or the causal structure quiz. These participants were excluded from analyses. Final analyses were conducted on 196 participants (128 F, 67 M) aged between 18 and 70 (*M* = 35.16). Power was estimated as 0.96 to find an effect size, Pillai *V* = 0.4.

#### Design and Materials

This study again drew influence from Rehder and Hastie (2004). Participants learnt about a category of ants known as a “kehoe.” As with Study 4, participants were randomly assigned to one of three causal conditions: Common Cause (*N* = 46), Common Effect (*N* = 50), or Causal Chain (*N* = 50), or a Control condition (*N* = 50). The four conditions were identical to Study 4, except participants only learnt about one category of ants (“kehoes”). After learning, participants were tested on their knowledge of the features associated with kehoes. Participants were presented with five questions, which each asked about one of the five features associated with the category. For each question participants were asked which of two characteristics was associated with the category (e.g., “Do kehoes have low or high levels of iron sulphate in the blood?”). The five questions were presented in a random order. Participants had two attempts to get every question correct before moving on to the next part of the study.

Next, participants in the three causal conditions also completed questions about the causal structure associated with kehoes. Participants were presented with four questions, corresponding to the four causal relationships they learned about in the first part of the study. Each question in the quiz asked participants to choose which of two statements correctly described the relationship between two features possessed by kehoes (e.g., “High levels of iron sulphate in the blood tends to cause a weakened immune system” or “A weakened immune system tends to cause a high level of iron sulphate in the blood”). Again, participants were given two attempts to get every question correct. The full list of quiz questions can be found in Supplementary Materials. Participants in the control condition skipped this part of the quiz and went straight to the concept appraisal phase.

The concept appraisal phase was similar to the previous studies. Participants completed a series of concept appraisal questions about the category they had just learnt about (see Table 4). To assess a fuller range of beliefs that may be associated with the category, in addition to the four questions from Study 4 at the top of Table 4, the next five questions in the table were about essentialism (Medin and Ortony, 1989; Haslam et al., 2000; Gelman, 2004) and then six additional questions were included that we hypothesised may vary according to causal condition. The fifteen questions were presented in a random order.

**TABLE 4:** Fifteen concept appraisal questions used in Study 5.

Induction	The scientists have discovered that when frightened some ants will run to the right and some ants will run to the left. Suppose that you observe three kehoes running to the left when frightened. How likely is it that another kehoe selected at random will also run to the left when frightened?	No more than chance–A lot more than chance
Informative-ness	How much do you think that knowing that a particular ant is a kehoe tells us about this particular ant?	Nothing at all–A great deal
How much you know	How knowledgeable do you think you are about kehoes?	Very unknowledgeable–Very knowledgeable
Explain	How confident do you feel about being able to explain kehoes to another person?	Very unconfident–Very confident
Naturalness	To what extent do you think that kehoe is a natural category rather than an arbitrary grouping?	Definitely arbitrary–Definitely natural
Mutability	How easy do you think it would be for an ant which has previously been classified as a kehoe to stop being a kehoe?	Extremely easy–Extremely difficult
Necessity	To what extent do you think kehoe is a category that has necessary features or characteristics?	Definitely does not have necessary features–Definitely has necessary features
Stability	To what extent do you think that kehoe is a category that has been stable over time?	Definitely has not been stable–Definitely has been stable
Discreteness	To what extent do you feel that kehoe is a clear-cut or fuzzy category?	Definitely clear-cut–Definitely fuzzy
Usefulness-1	Some categorisations are more useful than others. How useful do you think it is to categorise some ants as kehoes?	Not very useful–Very useful
Usefulness-2	For scientists hoping to control the populations of different species on the island, how useful is it to have learnt about kehoes?	Not very useful–Very useful
Realness	Do you think that scientists will agree that data gathered about kehoes will establish that they are a real category of ants?	Definitely yes–Definitely not
Education	Do you think residents of the island will be taught about kehoes in school?	Definitely yes–Definitely not
Variance	Do you expect kehoes to vary a lot in colour?	Definitely yes–Definitely not
Investment	Do you think it would be worthwhile for scientists to invest more time and money studying kehoes?	Definitely yes–Definitely not

#### Procedure

To begin with, participants were presented with the same backstory about the fictional category of ants presented in Studies 3 and 4. When participants turned the page, they were shown a table of the prototypical feature of the fictional category and were given as much time as they needed to study the table. Next, as in Study 4, participants in the three causal conditions were presented with further information about the causal relationships between the five features. Next, participants completed the quiz section of the study. After participants had successfully answered all of the questions (being excluded after two failed attempts), they completed a series of concept appraisal questions. The study was completed on Qualtrics.

### Results

**FIGURE 7:**
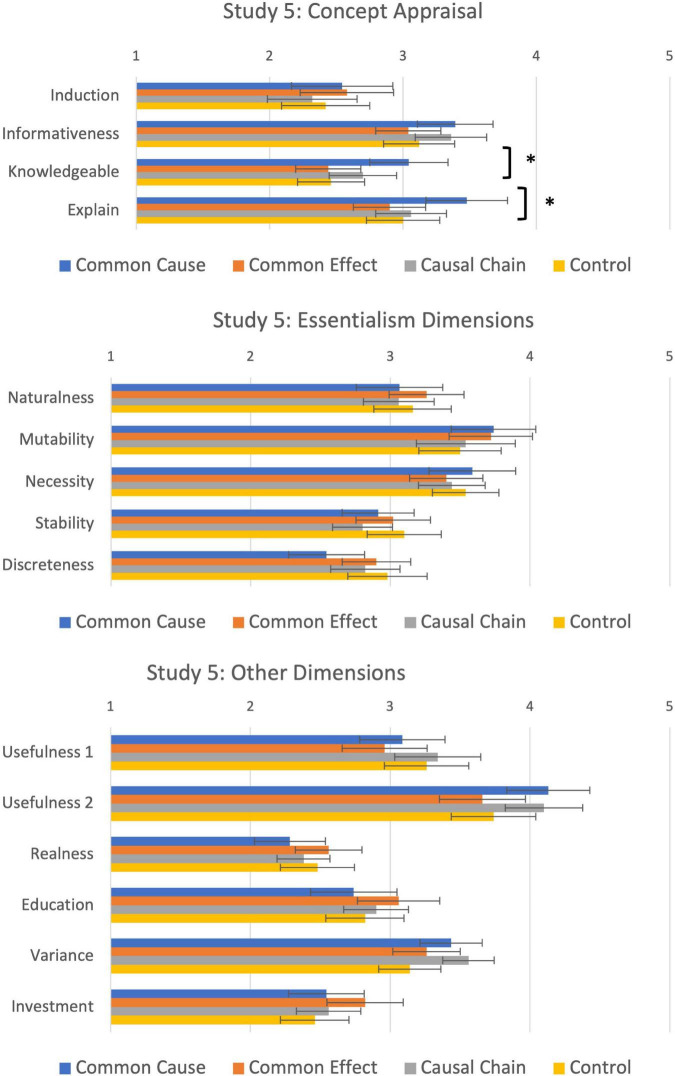
Dimensions of concept appraisal, essentialism and other dimensions as a function of causal condition in Study 5. *Significant at *p* < 0.05.

The focus of this study was on the fifteen concept appraisal questions. Mean judgements are shown in Figure 7. A between-subjects MANOVA of the effect of Condition on all 15 evaluation questions revealed that there were no significant differences overall between the four causal conditions on the concept appraisal questions [λ = 0.73, *F*(45, 530) = 1.33, *p* = 0.08, η_p_^2^ = 0.101]. To further explore planned effects on concept appraisal, two additional analyses were conducted looking at the effect of the manipulation first on the four original dimensions of concept appraisal, and second on the five questions relating to essentialist beliefs about the category.

#### Concept Appraisal Questions

A between-subjects MANOVA analysis of the four concept appraisal dimensions revealed a main effect of causal condition [λ = 0.90, *F*(12, 514) = 1.68, *p* = 0.035, η_p_^2^ = 0.035; Figure 7, top panel]. Univariate analyses indicated the main effect was driven by significant differences between causal conditions in *How Much You Know* [*F*(3, 197) = 3.96, *p* = 0.009, η^2^ = 0.06] and *Explain* [*F*(3, 197) = 3.91, *p* = 0.01, η^2^ = 0.06]. Participants in the common cause condition (*M* = 3.00, SD = 1.02) felt that they knew more about the category than participants in common effect (*M* = 2.42, SD = 0.84; *p* = 0.003) and control conditions (*M* = 2.49, SD = 0.86; *p* = 0.009). Participants in this condition (*M* = 3.51, SD = 1.04) also felt more able to explain kehoes than participants in the common effect (*M* = 2.92, SD = 0.96; *p* = 0.005), causal chain (*M* = 3.04, SD = 0.94; *p* = 0.02), or control conditions (*M* = 3.14, SD = 0.94; *p* = 0.02). There were no differences between conditions in *Induction* (*p* = 0.51) and *Informativeness* (*p* = 0.34). These findings are similar to Study 4.

#### Essentialism Questions

A similar analysis tested whether the causal manipulation influenced perceptions of essentialism about the category (Figure 7, middle panel). A between-subjects MANOVA with the five essentialism questions was non-significant [λ = 0.08, *F*(15, 564) = 1.03, *p* = 0.42, η_p_^2^ = 0.03]. Univariate analyses were all non-significant, with Bayes Factors between 0.18 and 0.39 favouring the null hypothesis.

### Discussion

As in Study 4, Study 5 found an effect of causal structure. Participants felt they knew more about the new category in the common cause condition as opposed to the other causal conditions (common effect and causal chain) and the control condition. They also felt they could explain more about the category in the common cause condition as opposed to the common effect, causal chain, and control condition. We did not, however, find any effect of the common cause condition on raising subjects’ ratings of the reliability of the concept.

## General Discussion

In a previous study we found that people assess their concepts for their usefulness in their thinking process. They assess concepts for understanding, reliability, and as tools for communication. To show this we tested eight dimensions of concept appraisal and found that these dimensions were valid between subjects and ranged across many conceptual domains (Thorne et al., 2021). This was the first demonstration that concept-users reliably engage in various forms of epistemic appraisal of their concepts. Furthermore, this earlier study discovered evidence for the existence of an underlying psychological factor (“Sense of Understanding”) which accounts for variation across concepts in four dimensions of concept appraisal: how much information you have about the category, how accurate that information is, how well you can explain the category, and whether you can predict what other people have in mind when they talk about the category.

Our interest in concept appraisal stems in part from the hypothesis that the way people appraise a concept epistemically will be an important determinant of whether and why individuals abandon and replace certain concepts. Across a social group, this would give some insight into why the concepts we use change over time, for example during a scientific revolution. Conceptual change is also bound up with changes in cultural norms (most likely part cause and part effect). The project of “conceptual engineering” aims to effect changes to concepts in common use in order to further social and political aims. Yet little is known about how such changes can be carried out and what kinds of interventions are effective. Manipulations that affect concept appraisals, through changing concept-users’ Sense of Understanding of a concept, or their epistemic appraisals along other dimensions, offer one avenue for potential interventions on the repertoire of concepts in common use. The current study is the first, preliminary examination of whether manipulations that are known to affect other forms of metacognitive assessment can also affect concept appraisal.

We aimed to explore two main questions. First, we wanted to establish whether manipulations that are known to modulate metacognitive judgements could indeed have an effect on concept appraisal. Second, if so, we wanted to discover what type of manipulation would be effective: procedural manipulations based on a thinker’s subjective experience in processing information, or declarative manipulations that provide new information that can connect with the person’s beliefs and knowledge about the category. The rationale behind the exploration of these two questions connects the discovery of the existence of epistemic appraisals of concepts with the issue of how these dimensions could be manipulated (manipulations that might eventually play a role in changing concepts). We looked at a body of literature that shows that subjects’ epistemic evaluations of beliefs and judgements about categories could be manipulated by factors stemming from the way in which information is processed (fluency vs. disfluency) and by declarative knowledge about the categories.

Studies 1–3 used metacognitive manipulations of fluency and replicated known effects on metacognitive judgements, but yet showed no effect of fluency on concept appraisal. Studies 4 and 5 used manipulations of the causal structure of the categories and showed that a category’s having more causal structure has an impact on thinkers’ appraisal of how much they know about the category but not on their assessment of the reliability of the categories themselves.

More studies are needed to better understand this difference. One future direction of research is to try other metacognitive manipulations commonly used in the literature. A possible factor is familiarity which can be manipulated in different ways, such as pitting familiar against unfamiliar concepts and measuring participants’ appraisals. Another approach would consist in comparing epistemic evaluations of concepts in familiar and unfamiliar domains.

A second direction for future studies could explore the different aspects of concept appraisal separately. As already said, our previous study (Thorne et al., 2021) showed that subjects’ assessments of their concepts tend to cluster into two or three groups: judgements relating to how well they understand the concept and judgements about how reliable the concept is (plus judgements on how good the concept is as a communicative tool). In these studies, we tried to find a metacognitive manipulation for both of these clusters. Study 5 suggests that dimensions relating to concept understanding can be influenced by manipulations of the causal structure of the concept, whereas concept reliability cannot.

Future studies may focus on manipulations of the different dimensions of concept appraisal in isolation. It could be that we come to the same conclusion when exploring each dimension separately; in this case we might have reason to conclude that many metacognitive manipulations do not affect dimensions of concept appraisal (neither conjointly, as in the present study, nor separately, if this is what is found in future studies). Alternatively, we might find that different aspects of concept appraisal are sensitive to different types of metacognitive manipulations. It is very possible that any appraisal of concepts is difficult to affect by piecemeal manipulations, since concepts tend to be complex bodies of information that include beliefs, prototypical representations, and other pieces of knowledge clustered in a representation. We might also need to use a finer-grained way to manipulate concept evaluations, not only by studying each dimensions of concept appraisal separately, but also by disentangling the different aspects of a conceptual representation.

## Data Availability Statement

The raw data supporting the conclusions of this article will be made available by the authors, without undue reservation.

## Ethics Statement

The studies involving human participants were reviewed and approved by Psychology Research Ethics Committee, City, University of London. The patients/participants provided their written informed consent to participate in this study.

## Author Contributions

ST, JS, JQ-D, NS, and JH conceived and planned the experiments. ST carried out the experiments and wrote the first draft of the manuscript. ST and JH contributed to the analysis and the interpretation of the results. JS wrote the final version of the manuscript. All authors provided critical feedback, helped to shape the research, analysis, and manuscript, and approved the submitted version.

## Conflict of Interest

The authors declare that the research was conducted in the absence of any commercial or financial relationships that could be construed as a potential conflict of interest.

## Publisher’s Note

All claims expressed in this article are solely those of the authors and do not necessarily represent those of their affiliated organizations, or those of the publisher, the editors and the reviewers. Any product that may be evaluated in this article, or claim that may be made by its manufacturer, is not guaranteed or endorsed by the publisher.

## Footnotes

1 Power analyses were performed using G × Power 3.1.9.6 (Faul et al., 2007). All reported estimates of power were based on the GLM tests employed for testing the null for that study.

2 Rehder and Hastie (2004) found an effect of causal structure on the induction dimension. However, in our design we didn’t ask subjects to perform inductions on the newly learnt categories and instead asked a general question about the category being good for induction.
