# Supplementary material for: Is Concept Appraisal Modulated by Procedural or Declarative Manipulations?
Source: Front Psychol. 2022 Mar 22;13:774629. doi: 10.3389/fpsyg.2022.774629 (PMC8982259; doi:10.3389/fpsyg.2022.774629)
Supplement: Supplementary file 1 [file Table_1.DOCX]

**Appendix A1:** Study 1 Pretest

In Study 1 we conducted a pretest to ensure that the bird stimuli selected for this study had relatively equal within-family similarity. In this pretest, participants were shown 10 exemplars of birds from 12 different bird families and were asked to make a judgement about the similarity of each family of birds. The six families of birds which were given medium ratings were selected as stimuli for the main study.

**Pretest**

**Participants and Design.** Thirty-three participants (18 Female, 15 Male) recruited through Prolific Academic participated in this study in exchange for a small monetary award (Age 18-45; M_Age_ = 29.00). Of the participants included in this study, 10 (30%) indicated knowing ‘not very much’ about birds; 20 (61%) indicated knowing ‘a little bit’ about birds; two (6%) indicated knowing ‘a fair bit’ about birds; one participant (3%) indicated knowing ‘a lot’ about birds.

Ten exemplars from 12 bird families (used by Walheim et al. 2011) were selected from images of birds on [www.whatbird.com](http://www.whatbird.com). Participants completed similarity ratings for each of the 12 bird families (*Chickadees*, *Finches*, *Flycatchers*, *Grosbeaks*, *Jays*, *Orioles*, *Sparrows*, *Swallows*, *Thrashers*, *Thrushes*, *Vireos*, and *Warblers*). To ensure that all participants started from the same baseline, we included and additional two anchor questions at the beginning of the questionnaire. For the anchor questions, participants were presented with ten exemplars from two families of dogs (*hounds* and *terriers*). We selected exemplars so that one family of dogs was perceptually more similar than the other.

**Similarity**. For each family of birds, the following instructions appeared at the top of a page followed by images of the ten birds in the family:

Below are 10 examples of birds from one particular family. Please study each bird carefully and then make a decision about how similar these birds are to each other using the rating scale at the bottom of the page. Please base your judgements of similarity on the aspects of the birds and not the similarity of aspects of the image (e.g., what they are standing on).

Each participant rated the two families of dogs, and the 12 different families of birds on a scale of 0 (extremely dissimilar) to 100 (extremely similar). The two families of dogs were presented in the same order for each participant, but each family of birds was presented in a random order.

**Results**. The mean rating for each item was calculated. The exemplars we selected for the families of dogs were given ratings that corresponded with our similarly manipulation; hounds (M = 60.45, SD = 19.42) were rated as less similar than terriers (M = 80.18, SD = 14.86; *t* (32) = 5.80, *p* <.001, *d* = 1.02). The mean ratings for the 12 families of birds can be seen in Table 1. To ensure that exemplars in each family of birds were not too similar or dissimilar to each other, the six medium families were selected for use in the main study.

**Table 1.** Similarity ratings for the 12 families of birds.

| Bird Family | Similarity |
| --- | --- |
| Grosbeak | 56.24 (25.41) |
| Finch | 61.27 (18.79) |
| Warbler | 61.70 (19.80) |
| Thrush | 65.09 (18.46) |
| Swallow | 65.27 (19.98) |
| Sparrow | 67.85 (19.84) |
| Jay | 70.36 (20.22) |
| Vireo | 72.85 (15.15) |
| Oriole | 74.09 (13.15) |
| Thrasher | 77.09 (14.35) |
| Flycatcher | 79.39 (17.03) |
| Chickadee | 85.94 (10.85) |

**Appendix B1:** Backstory for Studies 3-4

Please imagine that scientists investigating different species of insects in the world have discovered the existence of two new species of ant located on the volcanic island of Kehoe in the Western Pacific Ocean near Guam. They have called these two species Kehoe Ants and Victoria Ants. Scientists studying these two species of ant have discovered a number of characteristics that distinguish them. In order to document these new types of ant, the scientists created a table of characteristics that individually observed ants in each of the two species has shown.

You are a student working with these two scientists.

**Appendix C1:** category structures (Studies 3-4)

The Category Structure for Kehoe Ants (Study 3)

| Exemplar | F1 | F2 | F3 | F4 | F5 | F6 |
| --- | --- | --- | --- | --- | --- | --- |
| 1 | 1 | 1 | 1 | 1 | 1 | 0 |
| 2 | 1 | 1 | 1 | 1 | 0 | 1 |
| 3 | 0 | 1 | 1 | 1 | 1 | 1 |
| 4 | 1 | 1 | 0 | 1 | 1 | 1 |
| 5 | 1 | 0 | 1 | 0 | 1 | 1 |
| 6 | 0 | 1 | 0 | 1 | 1 | 1 |
| 7 | 1 | 1 | 1 | 0 | 1 | 0 |
| 8 | 1 | 0 | 1 | 1 | 0 | 1 |
| 9 | 1 | 0 | 1 | 1 | 1 | 1 |
| 10 | 1 | 1 | 1 | 0 | 1 | 1 |
| 11 | 0 | 1 | 0 | 1 | 1 | 1 |
| 12 | 1 | 1 | 1 | 1 | 0 | 0 |

Note: A 1 indicates that the Exemplar had the Kehoe value for the feature, and a 0 indicates that it had the Victoria value. For each category, exemplars 1-8 were presented in the learning phase and exemplars 1-2, 5-6, and 9-12 were presented in the test phase. Victoria Ant exemplars had the same structure.

The Category Structure for Kehoe Ants (Study 4)

| Exemplar | F1 | F2 | F3 | F4 | F5 |
| --- | --- | --- | --- | --- | --- |
| 1 | 1 | 1 | 1 | 1 | 0 |
| 2 | 1 | 1 | 1 | 0 | 1 |
| 3 | 0 | 1 | 1 | 1 | 1 |
| 4 | 1 | 1 | 0 | 1 | 0 |
| 5 | 1 | 0 | 1 | 0 | 1 |
| 6 | 0 | 1 | 0 | 1 | 1 |
| 7 | 0 | 1 | 1 | 1 | 0 |
| 8 | 1 | 0 | 0 | 1 | 1 |
| 9 | 1 | 0 | 1 | 1 | 1 |
| 10 | 1 | 1 | 0 | 1 | 1 |
| 11 | 0 | 0 | 1 | 1 | 1 |
| 12 | 1 | 1 | 1 | 0 | 0 |

Note: For each category exemplars 1-8 were presented in the learning phase and exemplars 1-2, 5-6, and 9-12 were presented in the test phase. Victoria Ant exemplars had the same distortions.

**Appendix D1:** Quiz Questions for Study 5

**Feature Questions**

1. Do kehoes have low or high levels of iron sulphate in the blood?
2. Do kehoes have a weak and strong immune system?
3. Do kehoes have thin or thick blood?
4. Do kehoes have a short or long lasting flight response?
5. Do kehoes have a below average or above average body weight?

**Causal Questions**

**Common Cause**

Which of these two statements is true for kehoes?

| 1. | a) | High levels of iron sulphate in the blood tends to cause a weakened immune system. |
| --- | --- | --- |
|  | b) | A weakened immune system tends to cause a high level of iron sulphate in the blood. |
|  |  |  |
| 2. | a) | High levels of iron sulphate in the blood tends to cause the blood to thicken. |
|  | b) | Thicker blood tends to cause an increase in levels of iron sulphate in the blood. |
|  |  |  |
| 3. | a) | High levels of iron sulphate in the blood tends to cause a short-lasting flight response. |
|  | b) | A short-lasting flight response tends to cause an increase in levels of iron sulphate in the blood. |
|  |  |  |
| 4. | a) | High levels of iron sulphate in the blood tends to cause an increase in body weight. |
|  | b) | A high body weight tends to cause an increase in levels of iron sulphate in the blood. |

**Common Effect**

Which of these two statements is true for kehoes?

| 1. | a) | High levels of iron sulphate in the blood tends to cause an increase in body weight. |
| --- | --- | --- |
|  | b) | A higher body weight tends to cause an increase in levels of iron sulphate in the blood. |
|  |  |  |
| 2. | a) | A weakened immune system tends to cause an increase in body weight. |
|  | b) | A higher body weight tends to cause the immune system to weaken. |
|  |  |  |
| 3. | a) | Thicker blood tends to cause an increase in body weight. |
|  | b) | A higher body weight tends to cause the blood to thicken. |
|  |  |  |
| 4. | a) | A short-lasting flight response tends to cause an increase in body weight. |
|  | b) | A higher body weight tends to cause a short-lasting flight response. |

**Causal Chain**

Which of these two statements is true for kehoes?

| 1. | a) | High levels of iron sulphate in the blood tends to cause a weakened immune system. |
| --- | --- | --- |
|  | b) | A weakened immune system tends to cause a high level of iron sulphate in the blood. |
|  |  |  |
| 2. | a) | A weakened immune system tends to cause the blood to thicken. |
|  | b) | Thick blood tends to cause the immune system to weaken. |
|  |  |  |
| 3. | a) | Thicker blood tends to cause a short-lasting flight response. |
|  | b) | A short-lasting flight response tends to cause blood to thicken. |
|  |  |  |
| 4. | a) | A short-lasting flight response tends to cause an increase in body weight. |
|  | b) | An increase in body weight tends to cause a short-lasting flight response. |
